# Enhancing the Antifungal Efficacy of Fluconazole with a Diterpene: Abietic Acid as a Promising Adjuvant to Combat Antifungal Resistance in *Candida* spp.

**DOI:** 10.3390/antibiotics12111565

**Published:** 2023-10-26

**Authors:** Maria Gabriely de Lima Silva, Luciene Ferreira de Lima, Victor Juno Alencar Fonseca, Lucas Yure Santos da Silva, Ana Cecília Calixto Donelardy, Ray Silva de Almeida, Cícera Datiane de Morais Oliveira-Tintino, Anita Oliveira Brito Pereira Bezerra Martins, Jaime Ribeiro-Filho, Maria Flaviana Bezerra Morais-Braga, Saulo Relison Tintino, Irwin Rose Alencar de Menezes

**Affiliations:** 1 Laboratory of Pharmacology and Molecular Chemistry (LFQM), Department of Biological Chemistry, Regional University of Cariri (URCA), Crato 63105-000, Ceará, Brazil; gabriellyscience@gmail.com (M.G.d.L.S.); lucas.yure@urca.br (L.Y.S.d.S.); cecilia.donelardy@urca.br (A.C.C.D.); anita.oliveira@urca.br (A.O.B.P.B.M.); 2 Laboratory of Applied Mycology of Cariri (LMAC), Regional University of Cariri (URCA), Crato 63105-000, Ceará, Brazil; lucieneflima@ymail.com (L.F.d.L.); victorjuno5@gmail.com (V.J.A.F.); flaviana.morais@urca.br (M.F.B.M.-B.); 3 Laboratory of Microbiology and Molecular Biology (LMBM), Regional University of Cariri (URCA), Crato 63105-000, Ceará, Brazil; ray.almeida@urca.br (R.S.d.A.); datianemorais@hotmail.com (C.D.d.M.O.-T.); 4 Oswaldo Cruz Foundation (Fiocruz), Fiocruz Ceará, Eusébio 61773-270, Ceará, Brazil; jaime.ribeiro@fiocruz.br

**Keywords:** anti-*Candida* activity, abietic acid, fluconazole, antifungal resistance

## Abstract

The increasing antifungal resistance rates against conventional drugs reveal the urgent need to search for new therapeutic alternatives. In this context, natural bioactive compounds have a critical role in antifungal drug development. Since evidence demonstrates that abietic acid, a diterpene found in *Pinus* species, has significant antimicrobial properties, this study aimed to evaluate the antifungal activity of abietic acid against *Candida* spp and its ability to potentiate the activity of fluconazole. Abietic acid was tested both individually and in combination with fluconazole against *Candida albicans* (CA INCQS 40006), *Candida krusei* (CK INCQS 40095), and *Candida tropicalis* (CT INCQS 40042). The microdilution method was used to determine the IC_50_ and the cell viability curve. Minimum Fungicidal Concentration (MFC) was determined by subculture in a solid medium. The plasma membrane permeability was measured using a fluorescent SYTOX Green probe. While the IC_50_ of the drugs alone ranged between 1065 and 3255 μg/mL, the IC_50_ resulting from the combination of abietic acid and fluconazole ranged between 7563 and 160.1 μg/mL. Whether used in combination with fluconazole or isolated, abietic acid exhibited Minimum Fungicidal Concentration (MFC) values exceeding 1024 μg/mL against *Candida albicans*, *Candida krusei* and *Candida tropicalis*. However, it was observed that the antifungal effect of fluconazole was enhanced when used in combination with abietic acid against *Candida albicans* and *Candida tropicalis*. These findings suggest that while abietic acid alone has limited inherent antifungal activity, it can enhance the effectiveness of fluconazole, thereby reducing antifungal resistance.

## 1. Introduction

The genus *Candida* includes many fungal species capable of causing opportunistic infections with elevated mortality rates, thus representing a significant public health problem [1,2]. Notably, epidemiological data have shown that fungal infections caused by different *Candida* species have increased significantly in recent years [3]. While *Candida* species are common in the normal microbiota, under certain circumstances, such as cancer therapy and immunity-related diseases like AIDS, they can cause systemic infections, contributing to increased morbidity and mortality [4]. Estimating the global prevalence and incidence of candidiasis depends on geographical location. However, the most common cause of healthcare-related *Candida* infections worldwide is attributed to *Candida albicans*, *Candida tropicalis*, *Candida krusei*, and *Candida glabrata* [5].

*C. albicans* and *C. tropicalis* are both members of the human and animal microbiota, commonly found in the digestive and genital tracts. They exhibit high prevalence, especially in hospital environments, with a significant impact on neutropenic patients [6,7,8]. Invasive candidiasis, whether caused by *C. albicans* or *C. tropicalis*, can present complex pharmacological challenges and is associated with substantial mortality. Once it reaches the bloodstream, this condition can lead to sepsis [6,7,8]. The literature data show that this *Candida* species presents high antifungal resistance rates of 40–80% against azole compounds, including voriconazole and posaconazole [8], representing a major health problem, especially in the Asia-Pacific and Latin American countries [9]. Another notable member of this genus is the species *C. krusei* (teleomorph *Pichia kudriavzeveii*), a typical member of the human microbiota. However, this pathogen is frequently associated with opportunistic infections. Since the mucosal regions are the primary site of infection, esophageal and cutaneous candidiasis [10] are the most common diseases caused by *C. krusei*, mainly affecting immunocompromised patients. While presenting a moderate resistance profile, this species shows intrinsic resistance to fluconazole and echinocandins, which impairs these drugs’ therapeutic efficacy [10,11].

The development of drug resistance in *Candida* species is linked to genetic variability and innate biofilm formation. It is important to highlight that the primary mutational source in this evolutionary process arises from recurrent exposure to antimicrobial agents, leading to the emergence of the biochemical and genetic changes observed in resistant strains. [4]. The rates of acquired resistance among different classes of antifungal drugs increase in the following order: *C. albicans* (resistance profile < 5%), *C. krusei* (resistance profile < 7%), *C. tropicalis* (resistance profile 4–9%), *C. parapsilosis* (resistance profile 4–10%), *C. glabrata* (4–16%). Considering the epidemiological importance of candidiasis, the increasing rates of antifungal resistance observed in recent years might impact the mortality rates due to infection by *Candida* species [12,13].

Given the significant resistance to the most commonly used antifungals, such as fluconazole, the search for new drugs capable of directly inhibiting fungal growth or enhancing the activity of these drugs is necessary to improve the therapeutic arsenal against multidrug-resistant strains. Another critical alternative with significant benefits to pharmacotherapy is combination therapy. Consistent evidence has demonstrated that using lower doses of antifungal drugs combined with certain compounds results in increased antifungal effectiveness [14,15]. Compounds that can increase the intracellular concentration of conventional antifungal drugs can favor its interaction with the molecular target, as observed in drugs whose mechanism of action involves the inhibition of ergosterol synthesis, modifications in ion homeostasis, damage to the cell wall organization and biogenesis, or interference on reproductive cell cycle and DNA double-strand break repair. In addition, combined therapy against antifungal-resistant strains has been shown to interfere with significant virulence factors such as biofilm production, morphological transition, and filamentous growth [16]. In this context, natural products are primary sources of bioactive compounds with the potential to be used in antifungal drug development [17].

**Figure 1:**
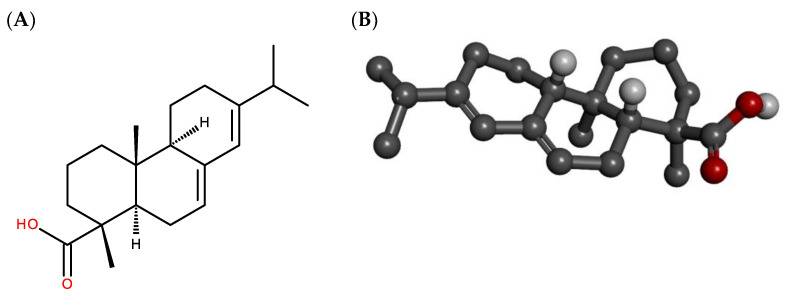
Chemical structure of abietic acid: 2D (**A**) and 3D (**B**).

Previous research has demonstrated that secondary metabolites of plants serve as a source of numerous molecules with the potential to treat fungal infections [14]. In this context, recent findings highlight resins as a substantial reservoir of bioactive compounds [17]. Resins are natural substances exuded by various plants, among which conifers such as *Pinus tabulaeformi*, *Pinus massoniana*, *Pinus palustris* Mill., *Pinus pinaster* Ait., *Pinus sylvestris* L., *Pinus laricio* Poiret, *Pinus longifolia* Roxb., *Pinus densiflora* Siebold et Zucc, and *Pinus thunbergii* Parlatore stand out as the most studied species [18]. Plant-derived resins consist of a mixture of terpenoids that have been used to treat inflammation and infections [19]. Abietic acid (Figure 1) is a diterpene formed by the oxidation process involving the CYP720Bs, a member of the cytochrome P450 complex, that catalyzes oxidation in the biosynthesis process to formations of abietadiene-like and resin acids (C18-acids) [20]. Studies characterizing the chemical profile of resins has identified abietic acid as one of the main constituents of these substances in various species of conifers [21]. Notably, previous research has revealed that this diterpene has biological activities that are antiparasitic [22], anti-inflammatory, and antibacterial [23], in addition to acting as a lipoxygenase inhibitor [24], which may imply anti-inflammatory activity [25,26].

The biosynthesis of ergosterol, a major sterol found in the cell membrane of many fungi, relies on the Lanosterol 14α-demethylase enzyme found in the endoplasmic reticulum of different cell types. This enzyme is a member of the cytochrome P450 family and has been used as a strategic target for rational design for new compounds that can be utilized in the treatment of a variety of fungal infections [25,27]. The literature data show that mutations on this enzyme are involved with mechanisms that confer antifungal resistance or cross-resistance to azole drugs [26,28,29].

Considering the need to discover new antifungal agents that are both safe and economically viable, and given the evidence of a cytochrome P450-mediated bioactivity of abietic acid, this study aimed to evaluate its antifungal activity against *Candida* spp. and analyze its interaction with the enzyme lanosterol-14α-demethylase using an in silico approach as a strategy of action mechanism investigation.

## 2. Results

The Minimum Fungicidal Concentration (MFC) analysis demonstrated that none of the tested concentrations of abietic acid inhibited fungal growth. In addition, the Minimum Fungicidal Concentration (MFC) values for these substances, as determined for the strains CA INCQS 40006, CK INCQS 40095, and CT INCQS 40042, were all above 1024 μg/mL. These findings suggest that even at the highest concentrations tested in this study, the fungicide activity of these substances is inefficient. Nevertheless, the highest concentrations achieved a slightly better fungistatic profile, reducing the growth of fungal strains but without clinical significance.

As can be observed in Table 1, abietic acid did not show clinically significant IC_50_ values, which was also observed for fluconazole against *C. albicans*. However, the abietic acid association increased fluconazole antifungal effect against CA INCQC 40006 and CT INCQS 40042. On the other hand, in experiments with the strain CK INCQS 40095, the combination of abietic acid with fluconazole reduced the inhibitory effect of the antifungal drug.

**Figure 2:**
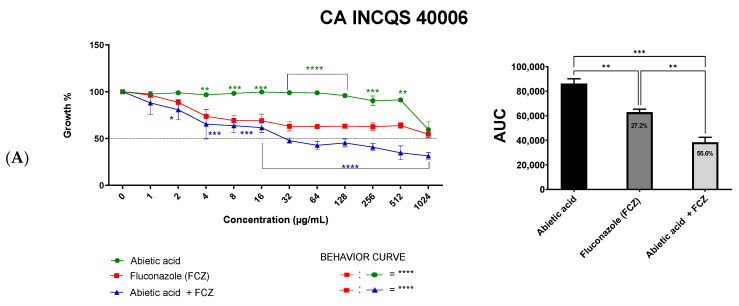

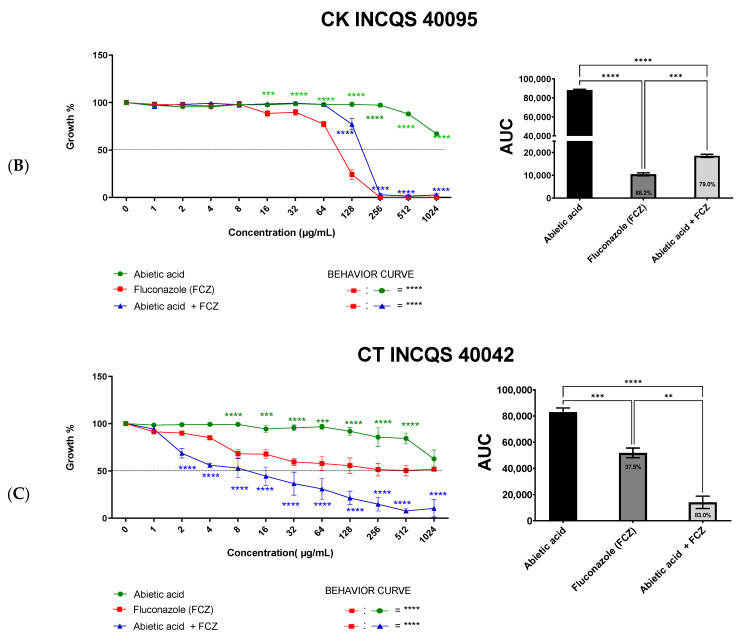
Growth curve of *Candida* spp. exposed to Abietic acid, Fluconazole (FCZ), and the combination of both: (**A**) *Candida albicans*: CA INCQS 40006; (**B**) *Candida krusei:* CK INCQS 40095; (**C**) *Candida tropicalis:* CT INCQS 40042. INCQS: National Institute of Quality Control in Health. These data are also represented as the area under the curve (AUC) showing the percent inhibition compared to abietic acid. The growth curve and AUC data were analyzed by an ANOVA followed by Tukey’s post hoc test; **** *p* < 0.0001; *** *p* < 0.001, ** *p* < 0.01. and * *p* < 0.05.

An analysis of the viability curve (Figure 2A–C) indicates the potentiation of the effect observed from the association of abietic acid and fluconazole against *C. albicans* (CA INCQS 40006) and *C. tropicalis* (CT INCQS 40042). On the other hand, the same association reduced fluconazole’s effectiveness against the CK INCQS 40095. An analysis of the area under the curve (AUC) (Figure 2A–C) showed that the combination of abietic acid and fluconazole resulted in significant modulation of activity against *C. albicans* (CA INCQS 40006) and *C. tropicalis* (CT INCQS 40042), increasing fungal growth inhibition from 27.2% to 55.6% and from 37.3% to 83.0%, respectively. However, in *C. krusei* (CK INCQS 40095) assays, discreet antagonism was observed, with growth inhibition changing from 88.2% to 79.0%.

The MFC analysis revealed that the *Candida* species used in this work presents a significant resistance profile against fluconazole and abietic acid. Nevertheless, the results observed in Figure 2, represented as the area under the curve (AUC; Figure 2), indicate a potentiation of antifungal activity against *C. albicans* and *C. tropicalis* that result from the association of abietic acid and fluconazole.

**Figure 3:**
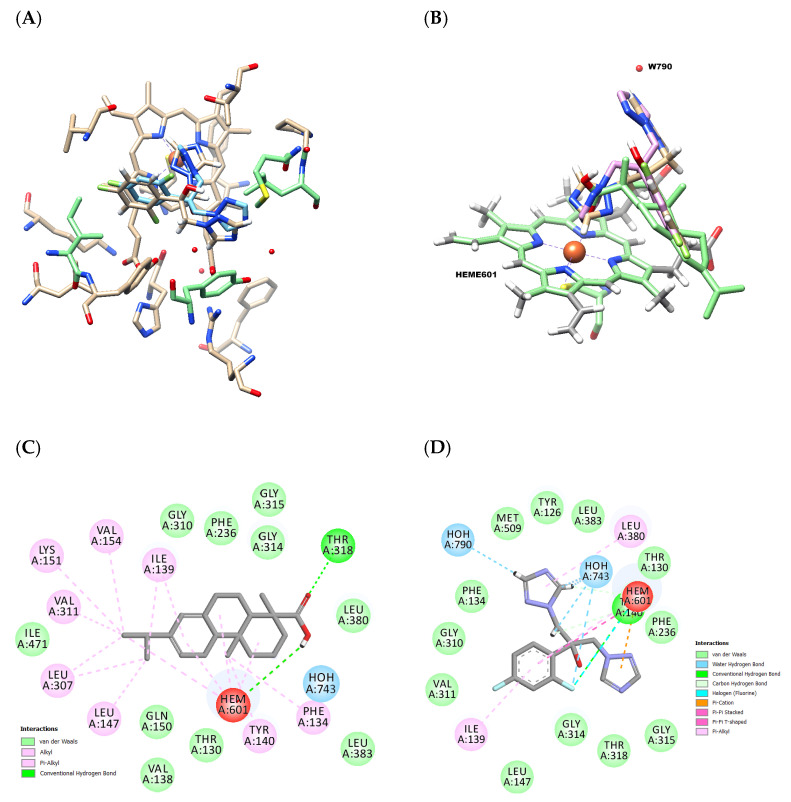
The three-dimensional observation of co-crystalized (blue color) and docked conformation (tan color) of fluconazole in the active region of lanosterol 14-α-demethylase (**A**) and binding mode of abietic acid (green color) and fluconazole (pink color) with the showing the coordination of N-2 with the heme group and hydrogen bond of N-4 with W790 (**B**). Two-dimensional (2D) residual inter-action diagram for abietic acid (**C**), fluconazole (**D**); interaction type is differentiated by colored circles (residues).

The biosynthesis of ergosterol, a major sterol found in the cell membrane of many fungi, relies heavily on lanosterol 14α-demethylase. This enzyme is highly similar to the lanosterol 14-α-demethylase found in *Saccharomyces cerevisiae* and *Candida species* [26,29]. Docking studies were conducted to investigate the interaction of abietic acid with the lanosterol 14-α-demethylase enzyme (PDBID:4wmz). Figure 3 displays the three-dimensional docked poses on the lanosterol 14-α-demethylase site (Figure 3A,B) and a two-dimensional interaction map (Figure 3C,D). Notably, the iron in the heme group creates four metal coordination bonds with the nitrogen atoms of porphyrin groups and a coordination with electrons of the nitrogen atom of fluconazole (Figure 3B) or oxygen in the hydroxyl group of abietic acid, which is positioned above the porphyrin plane. The distance between the nitrogen or oxygen atoms and the heme-liked iron are 2.2 and 3.25 Å, respectively. The hydrogen of the hydroxyl group of abietic acid establishes a hydrogen bond with nitrogen atoms of porphyrin groups (Figure 3C) with 1.7 Å. At the same time, fluconazole presents a hydrogen bonding network with water (W790 and W743) and the second triazole ring (Figure 3D). The superimposed poses show other hydrophobic interactions (which would be expected from the nature of the lanosterol substrate), such as van der Waals interactions between the triterpene ring with residues within 4 Å, including a total of ten residues and twelve residues with fluconazole showing similar interactions with residues Gly310, Gly314, Gly315, Phe236, and Leu383. The alkyl and π-alkyl interactions were observed in eight residues between the carbons of the triterpene rings and two residues to fluconazole, both presenting similar interactions with Ile139 residues (Figure 3C,D).

For the evaluation of the affinity of the ligand towards the active site, the binding affinity values (kcal/mol) and ligand efficiency of the best-scored pose were determined. The binding energy values of the compounds regarding the human CYP51 inhibition ranged from −8.2 (Ki estimated 975.8 nM and LE −0.37) to −8.3 kcal/mol (Ki estimated 824.3 nM and LE −0.38) to abietic acid and fluconazole, respectively, with the better negative value displaying promising binding. Furthermore, the differences in the binding energy observed can be explained by the interaction of the hydrogen bond network, which affects the positioning of the ligands and, subsequently, the drug binding. The key pharmacophores of typical CYP51 inhibitors consist of coordinated interaction with the iron present in the heme cofactor, the water-mediated hydrogen bonding network, and the hydrophobic interactions surrounding the pocket of CYP51. However, diverse side chains with different lengths could be accommodated in the active site of CYP51, contributing to a stabilized complex. As observed, the ligand binding pocket of the azole antifungal fluconazole (FCZ) has an additional affinity that is determining to better interactions with hydrophobic side-chains, the polypeptide backbone, and water-mediated hydrogen bond networks explained the high binding energy that observed in abietic acid.

**Figure 4:**
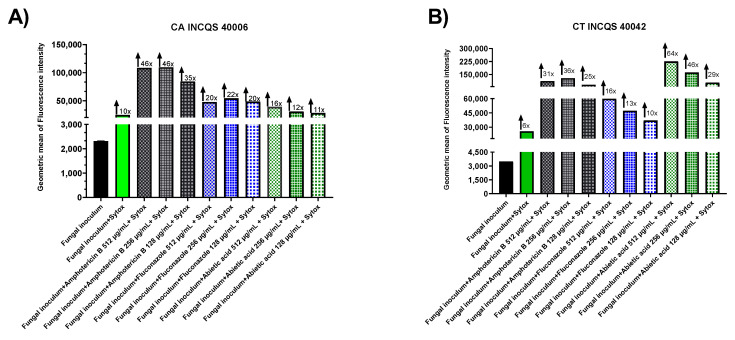
Membrane permeabilization induced by Amphotericin B, fluconazole, and abietic acid. (**A**) *Candia albicans* wild-type cells, and (**B**) *Candida tropicalis*. Sytox intensity was measured every minute for 24 min in a microplate fluorometer. Data represent the geometric mean ± SD of three individual experiments.

We tested the ability of amphotericin B, fluconazole, and abietic acid to cause interference in membrane permeability using Sytox Green as a fluorescent probe. Sytox Green increases its passage through the plasma membrane when it is structurally compromised and, inside, binds to nucleic acids to provide a fluorescent signal. In Figure 3, it can be seen that the fungus grown and associated with Sytox Green showed the lowest increase in fluorescence labeling; however, the addition of Amphotericin, a known antifungal that causes plasma membrane permeability by interaction with ergosterol in the fungal cell membrane causing the formation of pores, resulted in an increase in fluorescence by Sytox Green influx. The combination of Sytox Green with fluconazole also caused an increase in Sytox Green influx, indicating a decrease in membrane ergosterol, supporting the hypothesis that this action mechanism affects fungal growth and membrane permeabilization. We have observed that abietic acid has a similar effect against *C. albicans* (INCQS 40006; Figure 4A) and *C. tropicalis* (INCQS 40042; Figure 4B), corroborating with the docking results and the increased effect of fluconazole on the fungal growth assay supported by the hypothesis that this compound can promote a reduction in ergosterol synthesis by competitive inhibition of lanosterol 14-α-demethylase.

## 3. Discussion

The present study reports, for the first time, the capacity of abietic acid to positively modulate, in vitro, the antifungal action of fluconazole against *Candida* strains. Despite the absence of clinically significant intrinsic activity, abietic acid showed promising antifungal potential when combined with fluconazole against standard strains of *C. albicans* and *C. tropicalis.*

Literature reports show that terpenoids can enhance the action of antifungal drugs, as demonstrated by Himejima et al. (1992) using a phenanthrene diterpene. They demonstrated that abietane acid, found in the oleoresin of *Pinus* species, enhanced the activity of antifungal drugs against *Candida* strains [30]. In another study, plant extracts rich in diterpenes reduced the resistance of *C. albicans* to fluconazole [31,32]. Monico et al. (2017) reported that diterpenes can act as inhibitors of ABC and MFS transporters, which may partially contribute to their inhibitory effect on antifungal resistance [33].

Combined therapy using two or more substances with antimicrobial activity has emerged as a significant therapeutic strategy for reducing antifungal resistance, involving mechanisms such as efflux pump expression, mutations, deregulation of protein expression, and alteration of ergosterol biosynthesis [34]. Fluconazole is a triazole antifungal drug that acts by inhibiting cytochrome P450 enzymes, affecting the conversion of lanosterol to ergosterol, thus decreasing ergosterol biosynthesis, and leading to fungal membrane disruption. This membrane disruption enhances permeability and affects the activity of H^+^—ATPase. Additionally, evidence suggests that fluconazole may induce metabolic impairment by blocking its transcriptional regulator [35,36].

Although abietic acid was found to potentiate the action of fluconazole, when tested alone, it failed to inhibit the growth of the *Candida* species tested in this study. Similar results are found in the literature, as demonstrated by Lima et al. (2016), who showed that compounds derived from terpenic acid, gallic acid, and caffeic acid presented MIC values ≥1024 μg/mL against *C. albicans* ATCC 40042 and *C. tropicalis* ATCC 40006, indicating the absence of significant activity. However, their association with fluconazole, reduced the IC_50_ of the drug from 306.06 μg/mL to 67.38 μg/mL (gallic acid) and 109.12 μg/mL (caffeic acid), respectively [37]. Teodoro et al. (2015) also evaluated the antifungal activity of gallic acid against *Candida* species, finding no clinical relevance in the results. However, ellagic acid presented a significant fungistatic activity against *C. krusei* ATCC 6258 (MIC—125 µg/mL) [38]. Urzúa et al. (2008) showed that differences in the antimicrobial activities of diterpenes are related to structural aspects, such as the presence of hydrophobic regions and hydrophilic fragments that act as hydrogen bond donating groups [39].

Studies with compounds presenting chemical characteristics similar to those of abietic acid [40,41,42,43,44] showed that they have the ability to modulate the activity of antifungal drugs and highlighted the ability of these compounds to increase the sensitivity of Candida strains, which is consistent with the results of this study involving *C. albicans* and *C. tropicalis*. The amphiphilic nature of the chemical structure of abietic acid may explain this hypothesis, as it has the potential to interact with phospholipids leading to increased membrane fluidity and permeability. This process may facilitate the entry of fluconazole, which is consistent with the results obtained in this study. However, other studies have observed that abietic acid-like compounds may have opposite effects, reducing the sensitivity of fungi to antifungal agents [2,45]. Notably, evidence indicates that the sensitivity to these antifungal agents may vary depending on the fungus species [46,47].

In our tests, it was observed a significant difference in the effectiveness of fluconazole, inhibiting the growth of *C. krusei*, a species intrinsically more resistant to azoles than *C. albicans* and *C. tropicalis* [48]. This resistance does not imply genetic uniformity in the population of this species, suggesting the presence of variable resistance mechanisms. In fact, evidence indicates that some *C. krusei* cells may be sensitive to fluconazole, even in a dose-dependent manner [11]. This variability in resistance mechanisms also extends to the *C. albicans* and *C. tropicalis* species evaluated [49]. Individual susceptibility ultimately depends on the genetic composition of the microorganisms present in the colony used to prepare the inoculum for tests.

Lanosterol 14α-demethylase, also known as CYP51, is an enzyme that plays a crucial role in the biosynthesis of cholesterol-related lipids. This enzyme is a member of the cytochrome P450 superfamily, located in the endoplasmic reticulum of different cells. The inhibition of lanosterol 14α-demethylase has been used as a strategy for the treatment of fungal infections since this enzyme is also involved in the biosynthesis of ergosterol, a sterol found in the membrane of fungal cells. The inhibition of lanosterol 14α-demethylase can lead to the accumulation of toxic sterol intermediates that disrupt the structure and function of the fungal cell membrane, ultimately leading to fungal cell death [50]. The interactions observed between abietic acid and CYP51 support the key pharmacophores of typical antifungal inhibitors, which involves the coordination with and iron atom in the heme molecule of CYP51, the oxygen of hydroxyl stabilized by a hydrogen bond with the nitrogen of heme, and interaction with the hydrophobic cavity of CYP51 through the carbon rings of the skeletal structure of abietic acid, as observed in the crystal structures of lanosterol 14α-demethylase (PDB ID: 4wmz) [51]. By inhibiting this enzyme, triazole derivatives such as voriconazole or fluconazole disrupt ergosterol synthesis, leading to the accumulation of toxic sterol intermediates that disrupt the fungal cell membrane and ultimately lead to cell death [52]. There are several possible reasons for this opposite effect of abietic acid on the sensitivity of different *Candida* strains of fungi. Here are some of the most likely explanations:

(a) Differences in the genetic makeup of *Candida* strains: Different strains have different genetic backgrounds, which can affect their response to abietic acid. It is possible that the genes involved in the response to abietic acid are differentially expressed in different *Candida* strains, leading to different outcomes.
(b) Differences in the concentration and duration of abietic acid exposure: some strains may be more sensitive to abietic acid at lower concentrations or for shorter durations, while others require higher concentrations or longer exposure times to see an effect.
(c) Differences in the mechanisms of action of abietic acid: Abietic acid may affect *Candida* strains through different targets and mechanisms. For example, it may disrupt the fungal cell membrane, interfere with cellular signaling pathways, or cause interference with the enzymes involved in the biochemical process of synthesis of the cell wall. Some *Candida* strains may be more susceptible to one mechanism of action than others, leading to different outcomes.
(d) Interactions with other compounds: Abietic acid may interact with other compounds in the environment, such as antifungals of clinical relevance, other natural products, or synthetic drugs. These interactions could lead to synergistic or antagonistic effects, affecting the sensitivity of *Candida* strains.

Sytox Green is a compound that, while unable to traverse the membranes of intact live cells, readily penetrates compromised membranes and binds to nucleic acids, showcasing more than a 500-fold increase in fluorescence [53,54,55,56]. Amphotericin B, a recognized antifungal agent, induces permeability in plasma membranes, leading to pore formation (used as a positive control) [57]. Fluconazole, a primary antifungal medication known for its clinical effectiveness and low toxicity, acts by targeting the biosynthesis of ergosterol through the inhibition of lanosterol 14α-demethylase [58]. The literature data suggest a direct correlation between a reduction in ergosterol synthesis and increased membrane permeability [59]. Then, the results presented in this study indicate that the abietic acid’s primary mechanisms of synergism can be related to the inhibition of enzymes with crucial roles on fungal survival, causing the increase in the membrane’s permeability and interfering with intracellular ion homeostasis.

## 4. Materials and Methods

### 4.1. Drugs and Dilutions

Fluconazole was acquired from Globo Laboratory (São Jose da Lapa—Minas Gerais, Brazil); Abietic acid was obtained from Sigma-Aldrich Corporation (Spring, TX, USA), and Dimethyl sulfoxide (DMSO) was acquired from Merck, Darmstadt, Germany. A 0.02 g sample of abietic acid was weighed and diluted in 1 mL (20,000 μg/mL) of dimethyl sulfoxide (DMSO), forming the stock solution. Fluconazole was diluted in sterile water. The initial solutions of abietic acid and fluconazole were diluted with a culture medium to form a matrix concentration of 2048 μg/mL in test tubes or stored in Eppendorf tubes.

### 4.2. Microorganisms

The standard strains *C. albicans* INCQS 40006 (ATCC 10231), *C. tropicalis* INCQS 40042 (ATCC 13803), and *C. krusei* INCQS 40095 (ATCC 34135) were obtained from the Brazilian Institute of Quality Control in Health (INCQS), Oswaldo Cruz Culture Collection of the Oswaldo Cruz Foundation (FIOCRUZ).

### 4.3. Culture Medium

Sabouraud Dextrose Agar (SDA) and Sabouraud Dextrose Broth (SDB) were purchased from HIMEDIA^®^ (Maharashtra, India) and used according to the manufacturer’s instructions. The media were solubilized with distilled water and sterilized in an autoclave at 121 °C for 15 min.

### 4.4. Inoculum Preparation

All strains were initially maintained in SDA under refrigeration (8 °C). For the antifungal activity evaluation, the strains were cultured in SDA medium in a Petri dish at 37 °C for 24 h (overnight). Further, the microorganisms were prepared in tubes containing 4 mL of sterile saline solution (NaCl to 0.9%), and their turbidity was compared and adjusted to the 0.5 value on the MacFarland scale.

### 4.5. Minimum Inhibitory Fungicidal Concentration (MFC)

The MFC methodology was performed as described by Fonseca et al. (2022) [60] with adaptations on the final volume transferred from the microdilution plate for the Petri dish (10 μL) with a concentration that range of 0 to 1024 μg/mL. All tests were performed in quadruplicate. The growth of Candida colonies was analyzed 24 h after incubation at 37 °C. The MFC was defined as the lowest concentration capable of inhibiting colony growth [61,62].

### 4.6. Cell Viability Curve and Determination of the Half-Maximal Inhibitory Concentration (IC_50_)

In this assay, each well of a microdilution plate was filled with 100 μL of SDB. Then, 100 μL of solution of abietic acid or fluconazole, previously dissolved in DMSO, was added to the first well, and a serial dilution was performed to obtain concentrations ranging from 1024 μg/mL to 1 μg/mL. Subsequently, 10 μL (10% of the total solution) of the inoculum of the fungal strain was added, and the plates were incubated for 24 h at 37 °C. The readings were performed at 630 nm using a microtiter plate reader BioTek^®^ Cytation 1 (Agilent, CA, USA), and the results were used to obtain a cell viability curve and determine the IC_50._ Dilution and sterility controls were performed as previously described [63,64]. All tests were performed in quadruplicate.

### 4.7. Evaluation of Antifungal Activity Potentiation in Combination with Fluconazole

The analysis of antifungal activity potentiation was performed as previously described by Coutinho et al., (2008), with some modifications. Briefly, abietic acid, combined with fluconazole, was tested at a subinhibitory concentration based on the maximal value of the MFC. For those conditions when the MFC did not demonstrate clinical relevance (MFC values above 1024 μg/mL), the subinhibitory concentration is calculated as twice the maximal tested concentration (MC) (i.e., 2 × MC = 2 × 1024, so the subinhibitory concentration is 2048/8 = 256 μg/mL). The plates were filled with 100 μL of the abietic acid diluted in the medium. Serial microdilution was then performed by adding 100 μL of solution fluconazole to reach concentrations ranging from 1024 to 1 μg/mL. Subsequently, 10 μL of the microbial suspension was added (corresponding to 10% of the solution). Growth and dilution controls were also prepared. All tests were performed in quadruplicate. The plates were incubated at 37 °C for 24 h, and the readings were performed as described above [65,66,67,68].

### 4.8. Evaluation of Plasma Membrane Permeabilization

The permeability of the fungal plasma membrane was measured by Sytox Green uptake according to the methodology described by Mello et al. [69] with adaptations. The fungal inocula of *C. albicans* and *C. tropicalis* containing 1.5 × 10^8^ CFU were distributed in the 96-well black plate and treated with amphotericin B, fluconazole or abietic acid (128, 256 or 512 µg/mL) and then incubated for 1h; after this, 100 µL of Sytox Green was added at a final concentration of 1 µM and again incubated for 1 h. The uptake of SYTOX Green was quantified using a microtiter plate reader BioTek^®^ Cytation 1 (Agilent, CA, USA) equipped with a fluorescent filter with excitation at 485 nm and emission at 528 nm. Each treatment was performed in triplicate.

### 4.9. Molecular Docking Analysis

The crystal structures of lanosterol 14α-demethylase (PDB ID: 5tz1 and 4wmz) were obtained from the Protein Data Bank (https://www.rcsb.org/, accessed on 1 October 2023). For the docking operations, Autodock Vina was used, which required the receptor and ligands to be in pdbqt extension. Prior to docking, the enzymes were prepared with a pH of 7 ± 2. The M.G.L tools were used to prepare the enzymes, abietic acid, and the co-crystalized lead compound (fluconazole) in the proper format. The active site coordinates and volume were determined in a grid box with coordinates x:22, y:10, z:18, and x:20, y:20, and z:20 Å^3^, based on the cocrystal ligand coordinate. The estimated RMSDs between the co-crystallized and docked ligand were 1.0 Å (14α-demethylase), demonstrating the validity of the docking procedure. Visualizations were performed using Chimera v15 and BIOVIA Discovery Studio Visualizer v21.1.

### 4.10. Statistical Analysis

The data generated from the antifungal experiments were analyzed through one-way ANOVA followed by Tukey’s post hoc test, using GraphPad software version 6.0. For all analyses, the level of statistical significance was set at *p* < 0.05.

## 5. Conclusions

This study is the first to report the antifungal activity of abietic acid against standard strains of *C. albicans*, *C. krusei*, and *C. tropicalis*. Although abietic acid did not demonstrate a clinically relevant fungicidal action, its association with fluconazole promoted a relevant change in the ability of this antifungal to reduce the growth of fungal strains of *C. albicans* and *C. tropicalis* but not in *C. krusei*. Differences in the sensitivity of these species of *Candida* to abietic acid are likely due to a complex interplay of genetic, environmental, and chemical factors. However, further research is needed to determine the effectiveness of this compound against these *Candida* strains in vivo. Also, the mechanism of action of this compound can be better understood by evaluating its ability to inhibit virulence factors, morphological transition, and antifungal resistance in a biofilm model.

## Figures and Tables

**Table 1:** Half-maximal inhibitory concentrations (IC_50_) of antifungal agents against *Candida* strains.

Substance (IC_50_ μg/mL)	*C. albicans*	*C. krusei*	*C. tropicalis*
Abietic acid	1621 *	1748 *	2189 *
Fluconazole	1449.85 *	90.14	263.2
Abietic acid + Fluconazole	37.15	147.91	12.58

*Candida albicans*: CA INCQS 40006; *Candida krusei:* CK INCQS 40095; *Candida tropicalis:* CT INCQS 40042; INCQS: National Institute for Quality Control in Health. These values are expressed in μg/mL. * These values were calculated by interpolation of the curve equation.

## Data Availability

Data can be consulted upon request by contacting the corresponding author.
